# Exploring possible associations of the intestine bacterial microbiome with the pre-weaned weight gaining performance of piglets in intensive pig production

**DOI:** 10.1038/s41598-019-52045-4

**Published:** 2019-10-29

**Authors:** Xinghua Ding, Wensheng Lan, Gang Liu, Hengjia Ni, Ji-Dong Gu

**Affiliations:** 1 School of Biological Sciences, The University of Hong Kong, Pokfulam Road, Hong Kong, Hong Kong SAR China; 2 Shenzhen R&D Key Laboratory of Alien Pest Detection Technology, The Shenzhen Academy of Inspection and Quarantine. Food Inspection and Quarantine Center of Shenzhen Custom, 1011 Fuqiang Road, Shenzhen, 518045 China; 3 Hunan Province Key Laboratory of Animal Nutritional Physiology and Metabolic Process, Key Laboratory of Agro-ecological Processes in Subtropical Region, Institute of Subtropical Agriculture, Chinese Academy of Sciences, National Engineering Laboratory for Pollution Control and Waste Utilization in Livestock and Poultry Production, Changsha, Hunan 410125 China

**Keywords:** Microbiome, Microbiota

## Abstract

The pre-weaned weight gain is an important performance trait of pigs in intensive pig production. The bacterial microbiome inside the host is vital to host health and growth performance. The purpose of this study was to explore the possible associations of the intestinal microbiome with the pre-weaned weight gain in intensive pig production. In this study, several anatomical sites (jejunum, ileum, cecum, and colon) were examined for bacterial microbiome structure using 16S rRNA V4-V5 region sequencing with Illumina Miseq. The results showed that the microbial richness (estimated by Chao1 index) in jejunum was positively correlated with the pre-weaned weight gain. This study also revealed that the Firmicutes and Bacteroidetes in colon were the weight gaining-related phyla; while the *Selenomonas* and *Moraxella* in ileum and the *Lactobacillus* in both cecum and colon were the weight gaining-related genera for the pre-weaned piglets in intensive pig prodution. Several intra-microbial interactions within commensal microbiome correlated with the pre-weaned weight gain were excavated, as well. Overall, this study provides an expanded view of the commensal bacterial community inside four anatomical intestinal sites of the commercial piglets and the associations of the intestinal microbiome with the pre-weaned weight gaining performance in intensive pig production.

## Introduction

In the recent decades, the rapidly growing demand for pork has led to the intensive raising in pig production industry especially in China which already becomes the world’s largest producer of pigs^1,2^. And this commercial operation for pig production which concentrated populations of animals are housed can greatly enlarge the scale of raising number and improve the pig production rate^2^. In today’s intensive pig production, the pig’s litter size has become increasingly large due to the decades’ effort of selection for increasing the litter size. As the litter size has continuously become larger and larger, however, the average body size of the piglets born in the litter has decreased dramatically^3^. In the pig production, the smaller piglets at birth are always accompanied with the lower survival rates^4^. And smaller piglets at weaning presents many challenges such as lower survival rate, lower feed intake, lower daily gain, larger medication consumption, and longer raising period^5^. There is a theory in pig production industry that is a good weight gaining performance of piglets in pre-weaned period can benefit their subsequent daily gain and survivability, shorten their raising period, and decrease the operational cost and medication consumption of the pig producers^5,6^.

Intestinal bacterial populations are critically important in animal raising and production. Recently, studies examining the bacterial communities (often referred to as the microbiome) of production animals have increased dramatically with the advent of low-cost and high-throughput sequencing technologies^7,8^. Recent studies have been shown that the fecal microbiome was tightly associated with the health, the growth trait, and the feed efficiency in pigs^9–12^. However, a limitation of such studies that still remains is the collection of samples. There are also series studies reported that the bacterial communities inside the intestinal tract of the pigs^13–16^. But for the reason that commercial pigs in intensive operation are expensive to raise and are difficult to handle^8^, few studies have profiled the bacterial microbiome inside the commercial pigs in intensive operation and especially, much less is known regarding the commensal bacterial communities that exist across the intestinal tract and how these communities are associated with the important traits for intensive pig production such as the pre-weaned weight gaining performance.

Here, we sought to profile the bacterial community composition and diversity and explore the potential intra-microbial interactions among the commensal bacterial microbiome across four anatomical sites in the intestine of commercial piglets; and moreover, excavate the corresponding weight gaining-related bacteria and weight gaining-related intra-microbial interactions among the intestinal microbiome of pre-weaned piglets in the commercial intensive operation.

## Materials and Methods

### Animals, feeding, and sampling

All the experimental pigs were kept in a commercial intensive pig farm according to the farm’s manual for animal management. Samplings were carried out following Act on Welfare and Management of Animals as well as Guidelines for Animal Experiment. And this research was approved by the Animal Care Committee of the Institute of Subtropical Agriculture, Chinese Academy of Science. Thirty pregnant sows (Large White × Landrace) with parities of 2.5 ± 0.5 were randomly selected for this study. During the gestation period, the thirty pregnant sows were housed individually in gestation pen (2.6 m × 0.6 m) and then moved to the farrowing rooms (2.2 m × 1.5 m) on day 108 ± 2 of gestation. From day 90 of gestation to day 21 of lactation, the sows were fed with antibiotic-free perinatal formula diet twice a day (6:00 and 14:00) and free access to water. The composition of the perinatal formula diet is shown in Supplementary Table S1. During the suckling period, the piglets were not provided with supplemental feed. After weaning (at age of 21 days), one per each litter, thirty piglets in total, were randomly selected from these thirty litters for sampling. All the selected sows and piglets were monitored and recorded daily for general health conditions without any observed clinical signs such as diarrhea, lameness, necrosis, sneezing, inappetence, and so on. The parameters, the number of newborns and the survival rate of newborns, were used to assess the productive performance of the thirty selected sows. The birth weight and weaning weight of each selected piglet were carefully recorded. For sampling, the thirty piglets were sacrificed and the 5- to 10- cm sections, with the luminal contents included, of jejunum, ileum, cecum, and colon were tied off and stored at −80 °C until further processing.

### Sample processing and sequencing

From each sample, DNA was extracted from 0.25 g of sample by using QIAamp DNA Stool Mini Kit according to the manufacturer’s instruction (QIAGEN, German). The extracted DNA concentration and purity was monitored on 1.2% agarose gel electrophoresis. A sequencing library of the 16S rRNA gene V4-V5 regions was established according to the protocol provided by Illumina (https://support.illumina.com/content/dam/illumina-support/documents/documentation/chemistry_documentation/16s/16s-metagenomic-library-prep-guide-15044223-b.pdf). Phusion® High-Fidelity PCR Master Mix (ThermoFisher, United States) was used for two rounds PCR amplification. For the first round, Amplicon PCR (amplification of 16S rRNA V4-V5 region), the 515 F(5′-TTCCCTACACGACGCTCTTCCGATCT**GTGCCAGCMGCCGCGGTAA**-3′) and 926 R(5′-GAGTTCCTTGGCACCCGAGAATTCCA **CCGTCAATTCMTTTGAGTTT**-3′) primers^17,18^ were used following cycling parameters: one cycle of 94 °C for 2 minutes; followed by 25 cycles of 94 °C for 30 seconds, 56 °C for 30 seconds, and 72 °C for 30 seconds; and a final extension was performed at 72 °C for 5 minutes. The Amplicon PCR products were detected by 2% agarose gel electrophoresis and purified by AxyPrepDNA purification Kit (AXYGEN, USA). The 5 uL purified product of Amplicon PCR was used in a second round of PCR using forward (5′-**AATGATACGGCGACCACCGA**GATCTACAC-barcode-TCTTTCCCTACACGACGCTC-3′) and reverse (5′- **CAAGCAGAAGACGGCATACGA**GAT-barcode–GTGACTGGAGTTCCTTGGCACCCGAGA-3′) indexing primers^9^. The program for this Index PCR was the same as Amplicon PCR except that the cycle number was reduced to 8. The purification of Index PCR products was also conducted with 2% agarose gel electrophoresis coupled AxyPrepDNA purification Kit (AXYGEN, USA). The purified products were mixed at equal ratio and quantified via Qubit @ 2.0 Flurometer (Thermo Scientific, USA) and Q-PCR. At last, the library was sequenced on an Illumina Miseq 2 × 300 bp paired-end sequencing platform with MiSeq Reagent Kit v3 (600 cycles) (Illumina, USA) and 300 bp paired-end reads were generated.

### Sequencing analysis

Following sequencing, sequence reads were sorted by barcode to generate fastq files. And the Illumina MiSeq fastq reads were imported into the QIIME platform (v 1.8.0)^19^. Paired-end reads were assigned to samples based on their unique barcode and truncated by cutting off the barcode and primer sequence by Trimmomatic (v 0.36)^20^. FLASH (v 1.2.7)^21^ was applied to merge the paired-end reads into a complete read if they had a minimum overlap of 10 bp. In QIIME platform (v 1.8.0) quality controlled process^22^, the demultiplexed sequences were subject to the following quality filter: 200 ≤ length ≤ 580 bp; average quality score >25. High-quality reads were de-replicated into unique sequences and sorted by decreasing abundance, and singletons were discarded. And then, the filtered high-quality reads were compared with the reference database (Gold database, http://drive5.com/uchime/uchime_download.html) using UCHIME algorithm (UCHIME Algorithm, http://www.drive5.com/usearch/manual/uchime_algo.html)^23^ to detect chimera sequences (http://www.drive5.com/usearch/manual/chimera_formation.html). The chimera were removed and the effective clean sequences finally obtained^24^.

Operational taxonomic unit (OTU) picking was performed by Uparse software (Uparse v7.0.1001 http://drive5.com/uparse/)^25^ using all the effective clean sequences with clustering at 97% similarity and GreenGene (http://greengenes.lbl.gov/cgi-bin/nph-index.cgi)^26^ was based on RDP classifier (Version 2.2, http://sourceforge.net/projects/rdp-classifier/)^27^ algorithm to annotate taxonomic information at each taxonomic rank (Threshold: 0.8–1)^28^. The total observed OTUs, Chao1 index, and Shannon index which were used to assess the alpha diversity of each sample were calculated with QIIME platform (v 1.8.0)^22^.

### Statistical analysis

Because the intestinal bacterial microbiome is a commensal community in which many intra-mirobial interactions should exist resulted in the involved bacteria correlated with each other. Factor analysis was applied to excavate the intra-microbial interactions within the intestinal bacterial microbiome of the each anatomical site of intestinal tract. Factor analysis is a statistical method used to describe a group of correlated variables in terms of a potentially unobserved latent variable called factor. In this case, the observed variable means the abundance of each bacteria and factor (the latent variable) reflects the each potentially intra-microbial interaction. And then, the Spearman’s correlation analysis was performed to determine the correlation of the each observed variable and the each latent variable with pre-weaned weight gain of the piglets. In this study, the Spearman’s correlation analysis was also used to determine correlation among the sampling variables (the productive performances of the sows and growth performances of the piglets). All statistical analyses were carried out by SPSS (v 18.0) software.

### Accession number

The 16S rRNA gene sequence information in this study was deposited in NCBI Sequence Read Archive (SRA) under accession number: PRJNA566257.

## Result

### Growth performance of the piglets and productive performances of the sows

The sampling variables, including birth weight, weaning weight, pre-weaning weight gain of the piglets, parity of the maternal parent, number of newborns in the each litter, and survival rate of newborns in the each litter, are provided in Supplementary Table S2. As for the productive performance of the sows in this study, the number of newborns was found inverse correlated with the survival rate of newborns (r = −0.554, P = 0.001). And as for the growth performance of our selected piglets, the positive correlation of the pre-weaned weight gain vs. weaning weight (r = 0.922, P < 0.001) and the positive correlation of the birth weight vs. weaning weight (r = 0.464, P = 0.01) were found existed in this study [Table 1].

**Table 1:** Spearman correlation coefficients among different growth parameters of selected pre-weaned piglets and productive parameters of sows.

	Pre-weaned Weight Gain	Weaning Weight	Birth Weight	Survival Rate of Newborn	Number of Newborn
Weaning Weight	0.922***				
Birth Weight	0.140	0.464*			
Survival Rate of Newborn	−0.015	−0.092	−0.198		
Number of Newborn	−0.007	0.225	0.679***	−0.554**	
Parity of Sows	0.062	0.054	−0.046	0.159	−0.264

*,** and *** means the correlations are significant at p < 0.05, p < 0.01 and p < 0.001 levels, respectively.

### Overall intestinal microbiome structures in different intestinal sites of the pre-weaned piglets

**Figure 1:**
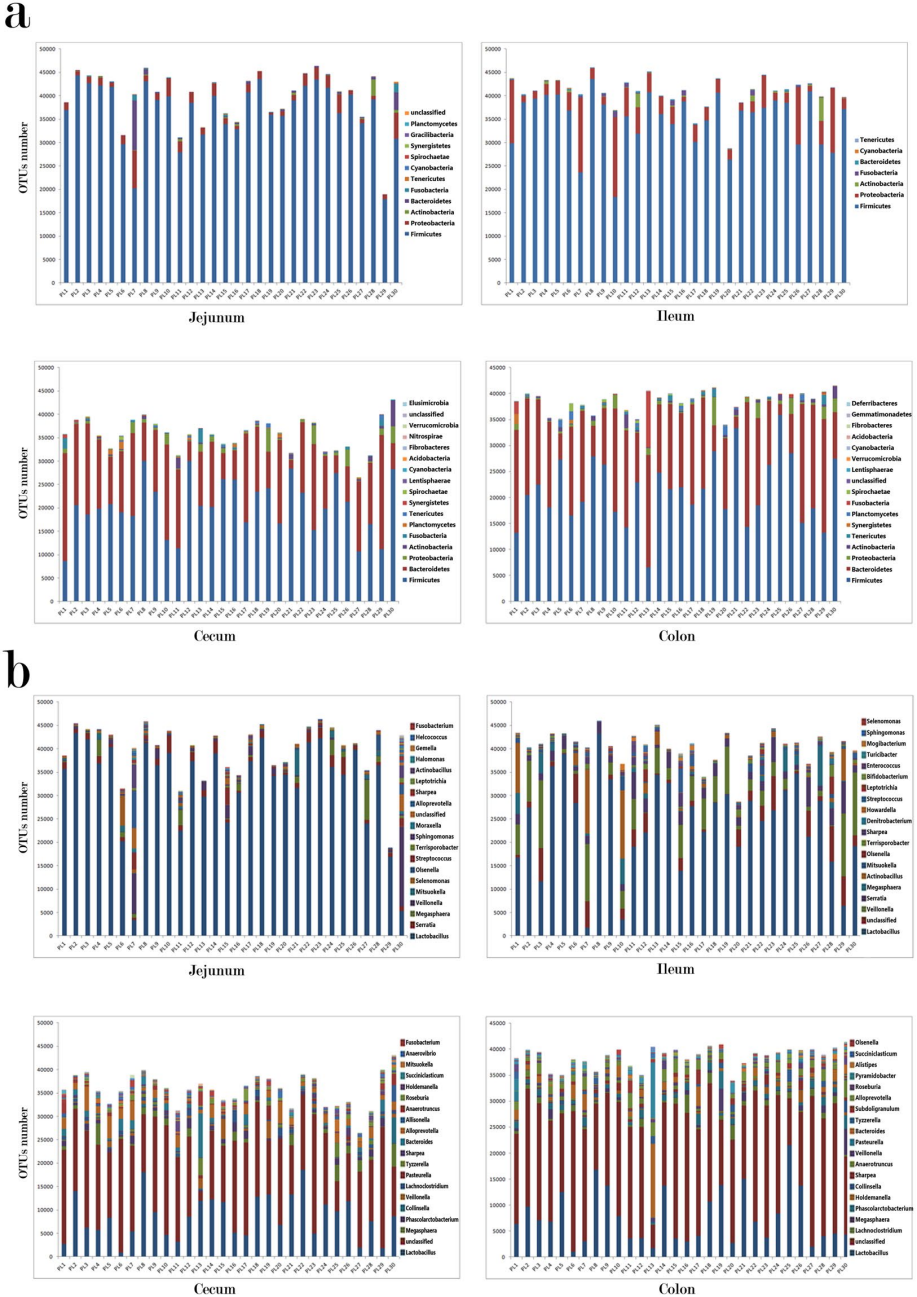
The OTUs abundances of the microbiome in different intestinal segments at the phylum level (**a**) and genus level (**b**); the legend only displays the top 20 abundant microorganisms.

From the 4 × 30 (4 intestinal segments for each piglet × 30 piglets in total) intestinal samples, a dataset consisting of 4652726 filtered classifiable 16S rRNA gene sequences ranging from 18909 to 45962 for all these samples was generated. And these sequences resulted in identification of a total of 1951 operational taxonomic units (OTUs) with ≥97% sequence similarity. We identified the taxonomic information and phylogenetic positions of these OTUs by aligning these OTUs sequences to the sequences in RDP Classifier Database with ≥97% sequence identity. Across all the samples, the Firmicutes and Bacteroidetes are the dominant phyla in the intestinal bacterial microbiome of all the 30 piglets. Specifically, the Firmicutes is the most dominant phylum in jejunum and ileum. The proportion of this phylum in these two intestinal sites is more than 70% for the most piglets. Although the microbiome in ileum of PL7 and PL10 show greater microbial richness and evenness than those of others, the proportion of the Firmicutes is still above 50%. As for the microbiome of cecum and colon, the Firmicutes and Bacteroidetes are the dominant phyla in all the 30 piglets. The average proportions of Firmicutes and Bacteroidetes in cecum are 47.18% and 30.20%; the average proportions of these two phyla in colon are 55.32% and 37.66%, respectively [Fig. 1a]. At the genus level, the *Lactobacillus* is dominant in jejunum for all the piglets in our study, but the proportions of the *Lactobacillus* are reduced gradually from the ileum, to cecum, till colon; corresponding to that, the microbial richness and evenness are considerably increased in these intestinal segments [Fig. 1b].

### Pre-weaned weight gaining-related observed variables of the intestinal microbiome

**Figure 2:**
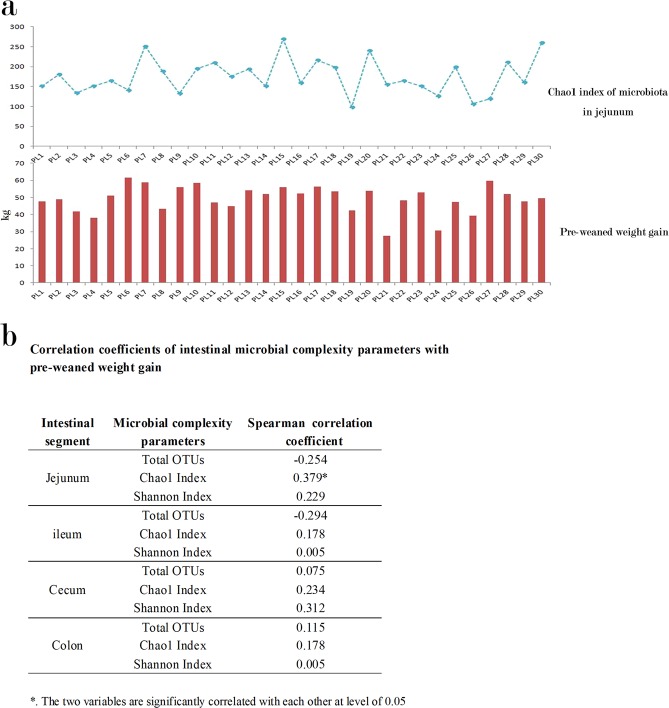
(**a**) The correlation between the Chao1 index in jejunum and the pre-weaned weight gain of piglets. (**b**) Correlation coefficients of the intestinal microbial complexity parameters in different intestinal segments with the pre-weaned weight gain. The total microbial abundance of microbiome is estimated by the index of the total OTUs; the microbial richness of microbiome is estimated by the Chao1 index; the microbial evenness of microbiome is estimated by the Shannon index.

**Figure 3:**
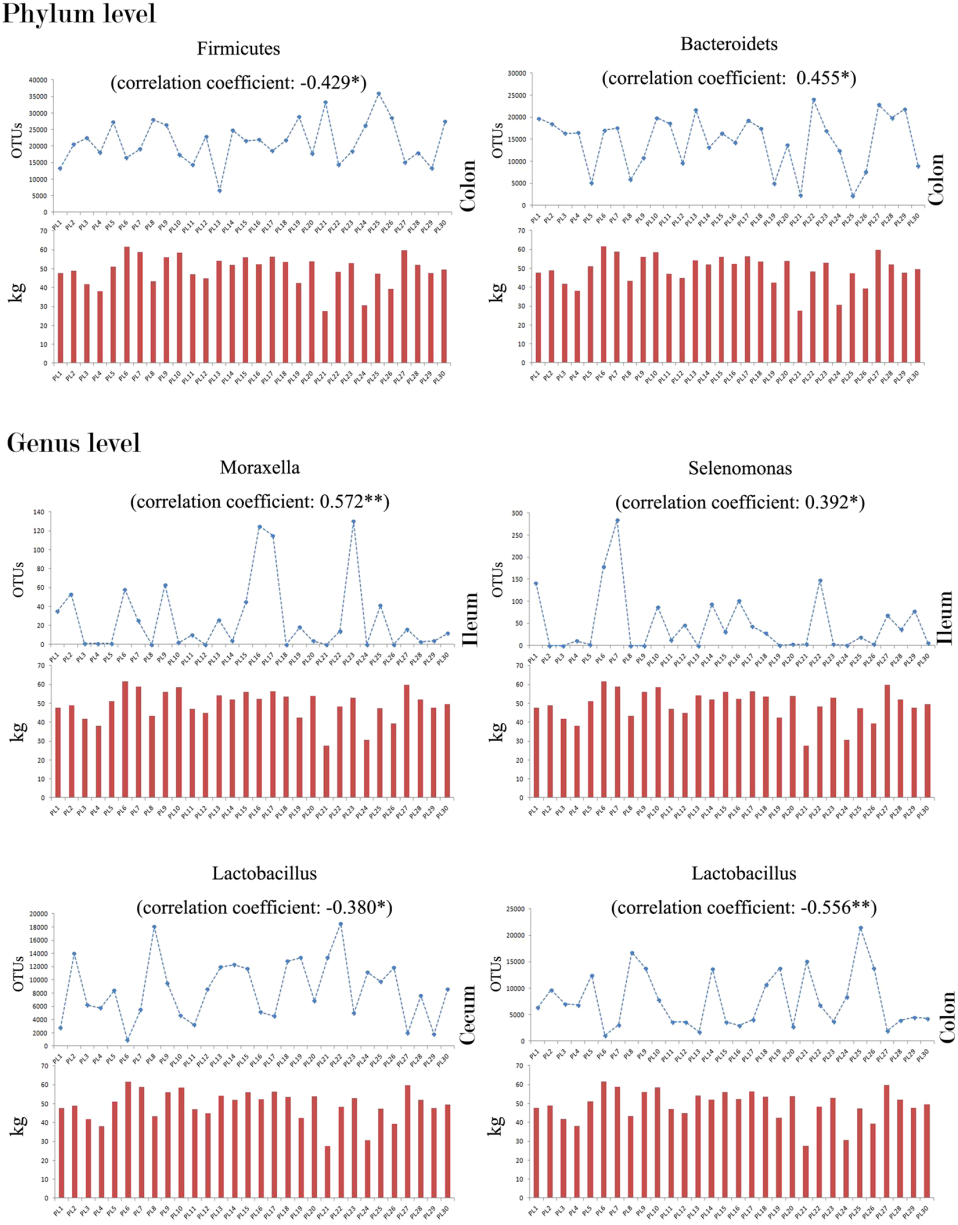
The correlations of the specific bacteria (observed variables) in different intestinal segments with the pre-weaned weight gain. The line charts (upper) are the abundances of the bacteria and the bar charts (below) are showing the pre-weaned weight gain of the piglets. *,** and *** means the correlations are significant at p < 0.05, p < 0.01 and p < 0.001 levels, respectively.

In our study, the alpha diversity indexes, including total observed OTUs, Chao1 index, and Shannon index were generated to assess the complexity of microbiome in each sample. The total microbial abundance of the microbiome was estimated by the total OTUs number; microbial richness was estimated by Chao1 index; microbial evenness was estimated by the Shannon formula. After our correlation analysis, the Chao1 index of jejunum was found positively correlated with the pre-weaned weight gain of piglets (r = 0.379, sig. = 0.039) [Fig. 2b] which means the piglets with higher microbial richness in their jejunum displayed the better pre-weaned weight gain performance [Fig. 2a]. In this study, the abundance of each bacterium constitutes the each observed variable in the microbiome dataset. When we explored phylum-level microbiome composition across all samples, our correlation test revealed that the abundances of the Firmicutes and Bacteroidetes in colon were correlated with the pre-weaned weight gain and the correlation coefficients were r = −0.429, P = 0.018 and r = 0.455, P = 0.011, respectively [Fig. 3]. As for the microbiome dataset at genus level, the abundances of of *Selenomonas* (r = 0.392, P = 0.032) and *Moraxella* (r = 0.572, P = 0.001) in ileum, and the *Lactobacillus* in both cecum (r = −0.380, P = 0.038) and colon (r = −0.556, P = 0.001) were correlated with the pre-weaned weight gain [Fig. 3].

### Pre-weaned weight gaining-related latent variables of the intestinal microbiome

**Figure 4:**
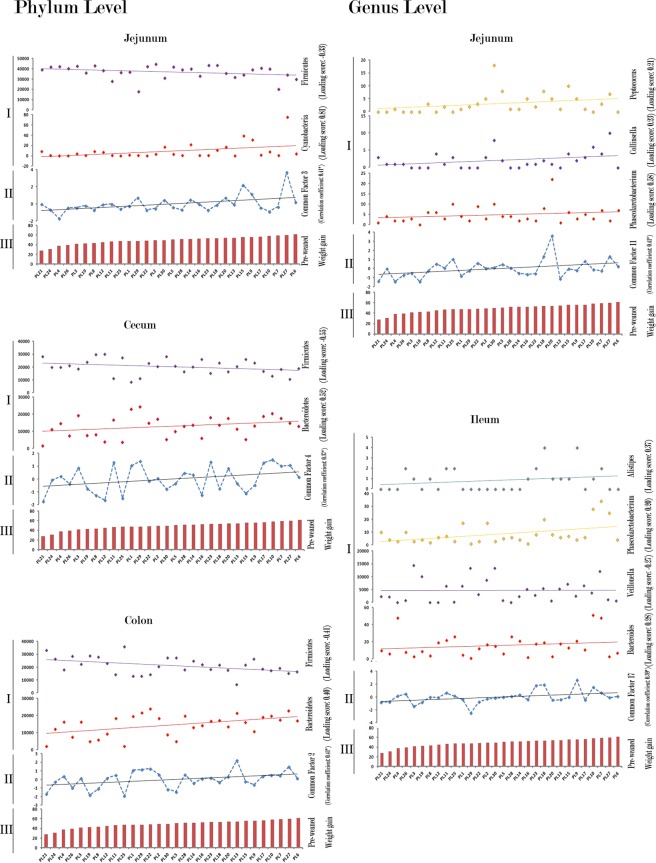
The correlations of the corresponding intra-microbial interactions (latent variables) in different intestinal segments with the pre-weaned weight gain. The intra-microbial interactions (latent variables) are presented as the factors (extracted by factor analysis) which represent the inner-correlations inside microbiome data-matrix for the intra-microbial interactions can result in the involved microorganisms correlating with each other. (**I**) the abundances of the correlated microorganisms in the corresponding intra-microbial interaction; (**II**) the factor (extracted by factor analysis) which represents the corresponding intra-microbial interaction; (**III**) the pre-weaned weight gain (kg) of the piglets. *,** and *** means the correlations are significant at p < 0.05, p < 0.01 and p < 0.001 levels, respectively.

For the reason that the intestinal bacterial microbiome is an ecological community of commensal, symbiotic, and pathogenic bacteria residing in intestine, many important physiological processes conducted by the intestinal microbiome should be the consequence of a series of bacteria working together. And these intra-microbial interactions can certainly result in the abundance of the involved bacteria correlated with each other. As a statistical method used to describe the correlated observed variables in terms of an unobserved latent variable called factor, factor analysis is suitable for analyzing the intestinal bacterial microbiome dataset to excavate the latent variables hiding behind our intestinal bacterial microbiome dataset. In this case, the each excavated factors by factor analysis represents a potential intra-microbial interaction in which a group of correlated bacteria involved. Thus, through factor analysis towards our microbiome data-matrix at phylum level, we excavated 4 factors inside the microbiome in jejunum, 4 factors in ileum, 7 factors in cecum and 7 in colon. And in these excavated factors, the 1 factor in jejunum, 1 factor in cecum, and 1 in colon were found correlated with the pre-weaned weight gain performance. The correlated factor in jejunum is the representative of the inverse relationship between the Cyanobacteria and Firmicutes; and the other two correlated factors in cecum and colon are both the representative of the inverse relationship between the Firmicutes and Bacteroidetes [Fig. 4]. Moreover, as for the factor analysis toward the microbiome at the genus level, there were 73 factors excavated in total (11 in jejunum, 18 in ileum, 21 in cecum, and 23 in colon, to be specific); and the two of them were found correlated with the pre-weaned weight gain. They are a representative of the intra-microbial interaction among the *Phascolarctobacterium*, *Peptococcus*, and *Collinsella* in jejunum and a representative of the intra-microbial interaction among the *Alistipes*, *Bacteriodes*, *Veillonella*, and *Phascolarctobacterium* in ileum, separately [Fig. 4].

## Discussion

The pre-weaned weight gain is an important performance trait of piglets in the intensive pig production, and it has already been used as an indicator trait of pig quality in this industry^6^. In this study, the birth weight vs. weaning weight displays correlation exited between these two growth performance parameters (r = 0.464, P = 0.01) supported the theory that the smaller piglets at birth will be accompanied with many adversity for the subsequent daily growth^3^. And the birth weight vs. pre-weaned weight gain shows nothing correlation existed between them. This result indicates that the piglets born with heavier birth weight cannot guarantee that they will have better pre-weaned weight gaining performance. In this study, the inverse correlation between the survival rate of newborn vs. number of newborn (r = −0.554, sig. = 0.001) was found which corroborates the statements claimed by Brans *et al*. (2018) and Salazar *et al*. (2018) that the decades of selection in the intensive pig production industry for increasing litter size has resulted in a too high stock density in each litter which may be a risk factor and may have the potential to intervene the growth of piglets^6,29^. Although a management practice, cross-fostering, has already been implemented to manage the large litter size, Brans CE still found that this management practice cannot benefit the pre-weaned weight gaining performance and bring heavier weaning weight of the piglets^6^. Therefore, obtaining the knowledge about the attribution of a causal link between the molecular biological physiology and the pre-weaned weight gain can aid producers to implement the corresponding management practices in enhancing the pig quality in the intensified pig production. Accumulating evidences suggest that intestinal microbiome plays a vital role in host health^9^. However, the knowledge about the commensal bacterial community inside the commercial pigs and the association of the microbiome with the important production traits such as the pre-weaned weight gain in intensive pig production is still limited.

There is a general awareness that a low microbial complexity in the intestine is considered detrimental for intestinal health as it is linked to many malnourished pathologies and disrupted weight gaining physiologies^30^. In this study, a correlation between the microbial richness (estimated by Chao1 index) in jejunum and the pre-weaned weight gain was found (r = 0.379, sig. = 0.039) [Fig. 2]. The intestinal microbial richness plays the important role in enhancing the adaptive potential of the intestinal microbiome, for the reason that lower microbial richness of intestinal microbiome could lead to the microbial simplification which harbors the risk of depriving the microbial gene pool of potentially useful intestinal gene reservoirs that allow adaptation to varied intestinal variables changes, such as temperature change, dietary ingredients change, and pathogen invasion^31^. Because the richness of the microbial gene pool can only be represented by the microbial richness (estimated by Chao1 index), there is a reasonable explanation why there are nothing correlations existed of others, the total microbial abundance (estimated by total observed OTUs) and the microbial evenness (estimated by Shannon index), with the pre-weaning weight gain in our study [Fig. 2b]. Therefore, the higher level of microbial richness involved in the stronger capability to maintain the homoestasis inside the jejunum segment should benefit the pre-weaned weight gaining physiology of piglets. Consdiering the jejunum is the primary segment responsible for the most nutrients absorption, its homestasis for the weight gaining physiology is self-evident.

And also in jejunum, a factor which represents the inverse relationship between the Cyanobacteria and Firmicutes was found positively correlated with the pre-weaned weight gain of piglets [Fig. 4]. Actually, these two phyla have similar metabolic function in intestine that they both take part in the anaerobic decomposition of diverse organic material by substrate-level phosphorylation to produce energy and release hydrogen gas that can be consumed by other microorganisms^32^. As the microbial richness enriched in jejunum of the piglets with higher pre-weaned weight gaining performance, it is reasonable that the Firmicutes, the most dominant phylum (accounts for more than 70%) in jejunum, should decrease and the Cyanobacteria should increase correspondingly to replace Firmicutes for performing the anaerobic decomposition of diverse organic material and releasing hydrogen gas for satisfying the hydrogen gas consumption of other microorganisms. At the genus level, a representative of the intra-microbial interaction among the *Phascolarctobacterium*, *Peptococcus*, and *Collinsella* in jejunum was found positively correlated with the pre-weaned weight gain, as well [Fig. 4]. The *Collinsella* and *Peptococcus* are well-proved hydrogen gas producing bacteria^33,34^; the *Phascolarctobacterium* is the important hydrogen gas consumer in intestine utilizing the hydrogen gas to succinate fermentation^35,36^. And the main end product of this succinate fermentation, Propionate, produced by the *Phascolarctobacterium* in intestine^35,37^, is a well-known precursor for gluconeogenesis, liponeogenesis and protein sysnthesis of the host when it’s absorbed by intestine and goes inside the body of host^38^. Therefore, we also surmise that the hydrogen gas producers in the microbiome may be interacted with the succinate fermentating bacteria at the jejunum segment to produce propionate which is important metabolic precursor for gluconeogenesis, liponeogenesis and protein synthesis of the host. And this bacteria-mediate metabolic process in jejunum may also involve in pre-weaned weight gain physiology of the piglets.

Next to the jejunum, as the intestinal luminal contents increased in the ileum, cecum, and colon segments, the microbial richness and microbial evenness in these intestinal segments were enriched and showed nothing correlations with the pre-weaned weight gain. In the ileum, we found an excavated factor (unobserved latent variable) in our factor analysis, which represents the intra-microbial interaction among the *Alistipes*, *Bacteroides*, *Veillonella*, and *Phascolarctobacterium*, was correlated with the pre-weaning weight gain [Fig. 4]. For the *Alistipes*, *Bacteroides*, and *Phascolarctobacterium*, although they can use simple sugars when they are available, the main sources of energy for the bacteria of these genera actually are complex indigestible glycans (such as host-derived complex polymers: N-glycan) in small intestine, where they play the fundamental role in processing of complex molecules to simple ones to make the absorption of those digestive-resistant polymers easier^39^. As these three genera increased in the ileum segment, the well known lactate specific fermentation genus, *Veillonella*^40^, decreased correspondingly in this intra-microbial interaction. Moreover, for the observed variables in our microbiome dataset (the abundance of each bacterium constitutes the each observed variable in our microbiome dataset), the abundances of the well-proved saccharolytic genera in ileum segment, the *Selenomonas* and the *Moraxella*^41,42^, were also found positively correlated with the pre-weaned weight gain and this finding further strengthen our surmise about the role that the microbiome in ileum segment plays in the pre-weaned weight gain physiology of piglets.

Considering all fats, protein, and simple carbohydrate such as sucrose, lactose, and starch are digested and absorbed before reaching the large intestine (cecum and colon segments), the luminal contents in cecum and colon largely include the complex digestive-resistant polysaccharides and refractory host-derived carbohydrates. And we found the factors (latent variables) represented the inverse interaction between Firmicutes and Bacteroidetes in the microbiome of these segments both were correlated with the pre-weaning weight gain [Fig. 4]. Among the several emerging theories about the intestinal microbiota with the weight gain, the ratio of Firmicutes to Bacteroidetes is considered as a biomarker to indicate the weight gain performance. This theory can find support in series studies, such as this Firmicutes/Bacteroidetes (F/B) ratio is correlated with body mass index (BMI) and tends to decrease in stout healthy subjects compared to it’s in lean healthy subjects^38^. Since the F/B ratio decreased in both cecum and colon segments of the piglets with better pre-weaning weight gaining performance in our study, we hyposthesis that the metabolic function of the Bacteroidetes in the bacteria-mediated fermentation for the refractory carbohydrate degradation may optimize the uptake of energy in the large intestine (cecum and colon segments) and benefit the pre-weaned weight gaining performance of piglets. Except for the intra-microbial interactions, we also found that an observed variable, *Lactobacillus*, was inverse correlated directly with the pre-weaned weight gain [Fig. 3]. The *Lactobacillus*, the simple carbohydrates’ consumer and lactate producer in the gut, has been reported by cumulating studies that the level of *Lactobacillus* inside the host body is inversely associated with the weight gain especially among those with intaking of complex carbohydrates for its poor performance on indigestible components utilization to maximize the energy intake in the weight gaining physiology^43^. Thus, our study also suggested that the bacteria-mediated fermentation for the refractory carbohydrate degradation may optimize the uptake of energy in the large intestine (cecum and colon segments) and benefit the pre-weaned weight gaining performance of piglets.

In this study, we delineated the possible associations of the intestine bacterial microbiome with the pre-weaned weight gaining performance of commercial piglets in intensive pig production. The results of this study suggested the high level of microbial richness in jejunum, bacteria-mediate hydrogen gas metabolic physiology in jejunum, and bacteria-mediated fermentation for the refractory nutrients degradation in ileum, cecum and colon could benefit the pre-weaned weight gaining performance of piglets in intensive commercial pig production. In conclusion, this study expends our knowledge about the commensal bacterial community inside the commercial pigs and the associations of the microbiome with the pre-weaned weight gaining performance in intensive pig production.

### Ethics approval

This study was approved by the Animal Care Committee of the Institute of Subtropical Agriculture, Chinese Academy of Science. All the pigs in this study were kept in an intensive pig farm according to the farm’s manual for animal management. Samplings were carried out following Act on Welfare of Animals and Guidelines for Animal Experiment.

## Supplementary information

Supplementary Information

## Data Availability

The sequencing raw data in this study was deposited in NCBI Sequence Read Archive (SRA) under accession number: PRJNA566257. And the others data generated or analysed during this study are included in this article.
