# Supplementary material for: Exploring possible associations of the intestine bacterial microbiome with the pre-weaned weight gaining performance of piglets in intensive pig production
Source: Sci Rep. 2019 Oct 29;9:15534. doi: 10.1038/s41598-019-52045-4 (PMC6820744; doi:10.1038/s41598-019-52045-4)
Supplement: Supplementary file 1 — Supplementary Information [file 41598_2019_52045_MOESM1_ESM.pdf]

Supplementary Information

Exploring possible associations of the intestine bacterial microbiome with the pre-weaned weight gaining performance of piglets in intensive pig production

*Xinghua Ding[1], Wensheng Lan[2], Gang Liu[3], *Hengjia Ni[3], *Ji-Dong Gu[1]

[1] School of Biological Sciences, The University of Hong Kong, Pokfulam Road, Hong Kong SAR, China.

[2] Shenzhen R&D Key Laboratory of Alien Pest Detection Technology, The Shenzhen Academy of Inspection and Quarantine. Food Inspection and Quarantine Center of Shenzhen Custom, 1011 Fuqiang Road, Shenzhen 518045, China.

[3] Hunan Province Key Laboratory of Animal Nutritional Physiology and Metabolic Process, Key Laboratory of Agro-ecological Processes in Subtropical Region, Institute of Subtropical Agriculture, Chinese Academy of Sciences, National Engineering Laboratory for Pollution Control and Waste Utilization in Livestock and Poultry Production, Changsha, Hunan 410125, China.

*Corresponding authors: Hengjia Ni (nihengjia@isa.ac.cn), Ji-Dong Gu (jdgu@hku.hk), and Xinghua Ding (u3005522@connect.hku.hk)

Xinghua Ding and Wensheng Lan contributed equally in this study.

Table S1| Nutrient components of the perinatal formula diet

Ingredient	Content, %	Nutrient content	
Corn	58.67	Digestible energy (MJ/kg)	14.14
Wheat bran	5.00	CP (%)	17.70
Wheat flour	2.00	CF (%)	6.80
Soy oil	4.00	Lys (%)	1.15
Soybean meal	20.50	Met (%)	0.36
Gluten meal	3.00	Cys(%)	0.30
Fish meal	2.50	Thr (%)	0.74
Lysine	0.15	Ca (%)	0.87
Threonine	0.05	P (%)	0.64
Valine	0.10		
Antioxidants	0.03		
Premix *	4.00		
Total	100		

* Premix provides the following per kilogram of the diet: VA 15000 IU, VD 3200 IU, VE 50 IU, VK 4.0 mg, VB1 4.0 mg, VB2 20 μg, choline chloride 800 mg, Fe 120 mg, Cu 20 mg, Zn 112 mg, Mn 24 mg, I 0.5 mg, Se 0.4 mg.

Table S2| The productive performance of sows and the growth performance of selected offsprings

Litter ID	Parity of Sows	Number of Newborn	Survival Rate of Newborn	Piglet ID	Birth Weight (kg)	Weaning Weight (kg)	Pre-weaned Weight Gain (kg)
L1	3	16	0.69	PL1	13.35	61.15	47.8
L2	3	12	1.00	PL2	15.4	64.25	48.85
L3	3	9	0.89	PL3	10.9	52.7	41.8
L4	2	14	0.79	PL4	18.25	56.3	38.05
L5	2	11	0.91	PL5	13.6	64.6	51
L6	2	11	0.55	PL6	12.4	73.9	61.5
L7	3	6	1.00	PL7	10.3	69.1	58.8
L8	3	7	1.00	PL8	6.85	50.1	43.25
L9	3	7	1.00	PL9	10.6	66.75	56.15
L10	3	10	1.00	PL10	17.5	75.85	58.35
L11	2	5	1.00	PL11	7.4	54.3	46.9
L12	3	9	0.89	PL12	13.75	58.75	45
L13	2	16	0.81	PL13	16.4	70.6	54.2
L14	3	5	1.00	PL14	9.2	61.25	52.05
L15	3	11	1.00	PL15	16.45	72.55	56.1
L16	3	15	0.80	PL16	14.2	66.65	52.45
L17	2	13	0.85	PL17	15.55	71.85	56.3
L18	2	15	0.93	PL18	15.75	69.4	53.65
L19	2	13	0.92	PL19	13	55.3	42.3
L20	2	17	0.94	PL20	19.2	73.05	53.85
L21	2	12	1.00	PL21	7.05	34.6	27.55
L22	2	10	1.00	PL22	13.75	62.15	48.4
L23	2	10	1.00	PL23	12.65	65.7	53.05
L24	2	15	1.00	PL24	19.4	50.15	30.75
L25	3	14	0.93	PL25	18.6	65.85	47.25
L26	2	4	1.00	PL26	7.2	46.5	39.3
L27	2	9	1.00	PL27	15.05	74.85	59.8
L28	3	8	1.00	PL28	12.9	64.9	52
L29	3	10	1.00	PL29	18.25	66.05	47.8
L30	2	10	0.90	PL30	12.45	62.05	49.6
